# Leptospira Seropositivity in Humans, Livestock and Wild Animals in a Semi-Arid Area of Tanzania

**DOI:** 10.3390/pathogens10060696

**Published:** 2021-06-03

**Authors:** Georgies F. Mgode, Ginethon G. Mhamphi, Apia W. Massawe, Robert S. Machang’u

**Affiliations:** Pest Management Centre, Africa Centre of Excellence for Innovative Rodent Pest Management and Biosensor Technology Development, Sokoine University of Agriculture, P.O. Box 3110 Morogoro, Tanzania; mhamphi@sua.ac.tz (G.G.M.); massawe@sua.ac.tz (A.W.M.); machangu@sua.ac.tz (R.S.M.)

**Keywords:** leptospirosis in Africa, animal leptospirosis, goats, sheep, rodent and shrew reservoirs, leptospirosis burden, microscopic agglutination test (MAT), water-borne infection, zoonotic disease transmission, human-animal interaction

## Abstract

Background: Leptospirosis is among the major neglected zoonoses in developing countries. The prevalence of leptospirosis remains underestimated in many African countries because of limited diagnostic facilities. We studied *Leptospira* seropositivity prevalence in humans, sheep, goats and rodents in a semi-arid region of central Tanzania and compared findings with reports from humid tropical areas. The aims were to establish the disease burden in different settings; understand circulating *Leptospira* serovars and potential major reservoirs for establishing appropriate control measures. Methods: Humans, sheep, goats, rodents and shrews (insectivores) were sampled from Bahi district, a semi-arid area in central Tanzania. Samples were tested for leptospiral antibodies using microscopic agglutination test (MAT) consisting of *Leptospira* serovars mainly reported in Tanzania and reference strains. Findings were compared with previous data to determine the disease epidemiological patterns. Results and conclusion: Semi-arid area showed high *Leptospira* seropositivity prevalence in humans and domestic animals due to intensive human–animal interactions at scarce water points and by flash flooding which occur in the area. Rodent population in the semi-arid areas was relatively low due to flooding. *Leptospira* seropositivity in rodents was also slightly lower, and the rodents appeared to be prolific breeders, probably as a means to compensate for the lost population during extreme drought as well as during short spells of floods. Intensive human–animal interaction in the semi-arid areas especially, in water sources in valleys where human and animals often meet, likely increased the risk of leptospirosis transmission to rice farmers in the area. Goats and sheep which are kept around homesteads had higher leptospiral antibodies prevalence (62%), nearly double of the 38% reported in same species in humid tropical regions of Tanzania. Livestock, especially goats and sheep, could be the major source of leptospirosis transmission to humans. Vaccination of livestock with vaccines against local *Leptospira* strains should be encouraged, and rodent control emphasized, as part of a management strategy against leptospirosis. Public awareness of leptospirosis must also be raised and supported by availability of rapid test kits in clinics for preliminary testing of leptospirosis in people with fevers of unknown origin.

## 1. Introduction

Leptospirosis is a neglected zoonotic disease caused by spirochete bacterium of the genus *Leptospira*. *Leptospira* and leptospirosis disease is widespread but less known or only mentioned in public and medical communities in many African countries [1], unlike its popular sibling pathogens, namely *Treponema*, the causative agent of syphilis, and *Borrelia* that causes Lyme and relapsing fevers. Lack of obvious and distinctive symptoms in leptospirosis, unlike syphilis and Lyme disease, probably make leptospirosis less noticeable, although the syphilis and Lyme disease are also difficult to diagnose and are characterized by unspecific symptoms in the chronic phase. Leptospirosis is masked strongly by popular tropical diseases in developing countries. Leptospirosis in humans is mainly associated with symptoms such as fever, headache, jaundice, chills, vomiting and muscle pains [2]. These symptoms are also found in malaria, typhoid fever, dengue and other diseases. 

Human leptospirosis has been widely reported in select geographic regions of Tanzania [3,4,5,6,7,8]. Leptospirosis is also reported in rodents and domestic animals mainly from regions with rainy tropical climate in northern, eastern and southwestern Tanzania [8,9,10,11,12]. Leptospirosis information is lacking from the semi-arid central Tanzania [13] inhabited by agropastoralist communities with close human–animal interaction mainly through water points and housing. The area has flash floods that may favor leptospirosis transmission. 

We conducted a cross sectional *Leptospira* seropositivity surveillance in Bahi district, Dodoma region, central Tanzania, within the Bahi depression on the Eastern Rift Valley of Tanzania [13]. The valley and the lake located in this semi-arid area support agropastoralist communities engaged in livestock keeping and agriculture, including rice farming. The study objectives were to determine *Leptospira* seropositivity prevalence in humans, domestic small ruminants and rodents in the semi-arid land and within the wetland valley, which is the major source of water for agriculture, livestock and human consumption in this semi-arid setting. Furthermore, to compare *Leptospira* seropositivity prevalence and epidemiology of leptospirosis in semi-arid area versus tropical (non-arid, rainy) geographical regions of Tanzania. We also determined the common *Leptospira* serovars in the areas for establishing candidates for inclusion in the diagnostic antigen panel.

## 2. Results

### 2.1. Human Leptospira Seropositivity

Blood samples were obtained from 50 individuals from neighboring health facilities in Bahi. Fifteen individuals (30%) were positive for leptospiral antibodies. Thirteen of the seropositive individuals reacted against *L. kirschneri* serogroup Icterohaemorrhagiae serovar Sokoine, while three individuals were positive for *L. kirschneri* serogroup Grippotyphosa serovar Grippotyphosa. One of the three positive individuals against serogroup Grippotyphosa serovar Grippotyphosa cross-reacted with serogroup Icterohaemorrhagiae serovar Sokoine. *Leptospira interrogans* serogroup Australis serovar Lora reacted with one individual that was also positive for serovar Sokoine. *Leptospira interrogans* serogroup Pomona serovar Pomona and *L. borgpetersenii* serogroup Ballum serovar Kenya were not detected in humans. 

### 2.2. Rodents and Shrews

A total of 45 rodents and shrews (insectivores) were captured in the study area. The population of rodents during the study suggests a low population density, indicated by relatively few rodents captured in the study sites (*n* = 45) despite a large number of traps (200 traps) set per night. Low catch was observed particularly on rice fields and the river valley that had experienced floods during the rainy season that ended a month before trapping started in the dry season. Among the captured rodents were *Mastomys* species, of which two adult females were pregnant with a total of 23 and 25 embryos, respectively, compared to the average number of 12 to 13 embryos commonly recorded for this species in Tanzania (unpublished data). Leptospiral antibody seropositivity was relatively low in rodents, whereby 7 out of 45 captured rodents tested positive (15.5%). 

*Leptospira* antibodies to *L. kirschneri* serovar Sokoine were detected in all seven positive rodents.

### 2.3. Goats and Sheep

Leptospiral antibodies were detected in 28 out of 45 (62%) goats using MAT. Twenty-six (92.8%) of the 28 positive goats reacted with *L. kirschneri* serogroup Icterohaemorrhagiae serovar Sokoine. Two of the 26 positive goats against serovar Sokoine were also positive (cross-reacted) against *L. kirschneri* serogroup Grippotyphosa serovar Grippotyphosa. *Leptospira borgpetersenii* serogroup Ballum serovar Kenya and *L. interrogans* serogroup Pomona serovar Pomona were demonstrated in one animal each, making 28 the sum of positive goats. Antibodies against four of the five *Leptospira* serovars used in MAT was thus detected in goats. *Leptospira interrogans* serovar Lora was not detected in the tested goats. 

Leptospiral-positive sheep were 34 out of 56 (60.7%). Twenty-three sheep out of the 34 (67.6%) positive animals reacted with *L. kirschneri* serovar Sokoine, while *L. kirschneri* serovar Grippotyphosa antibodies were found in 14 (35%) of the 34-positive sheep. *Leptospira interrogans* serovar Lora reacted with one serum sample that was also positive for serovar Grippotyphosa. *Leptospira borgpetersenii* serovar Kenya did not react with any of the tested sheep serum samples.

**Figure 1:**
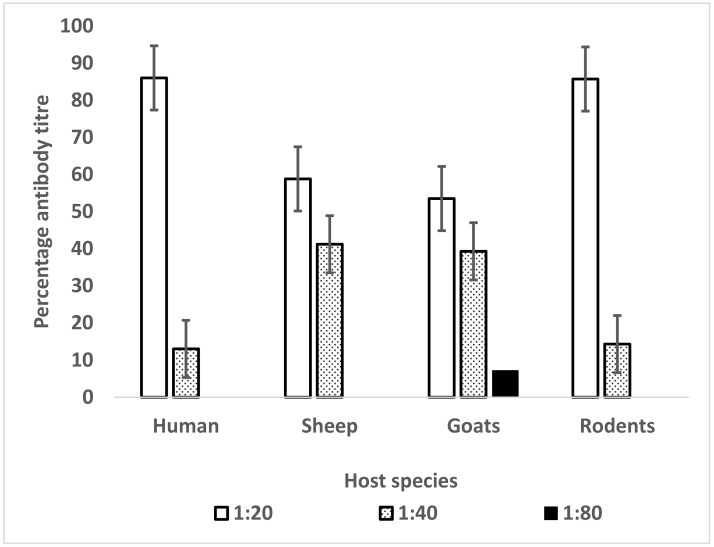
Proportion of leptospiral antibody titers in different host species. Higher titer 1:80 was obtained in two goats against *Leptospira* serovar Grippotyphosa and serovar Sokoine.

The composition of leptospiral antibody seropositivity in each host species is presented in Table 1 and Figure 1.

The proportion of leptospiral antibodies in rodents was 15.5%. Overall, the majority of the MAT titers found in the reactive samples after titration from 1:20 to 1:20,480 were low for all species tested, indicating potential endemism of leptospirosis in the study area. 

**Figure 2:**
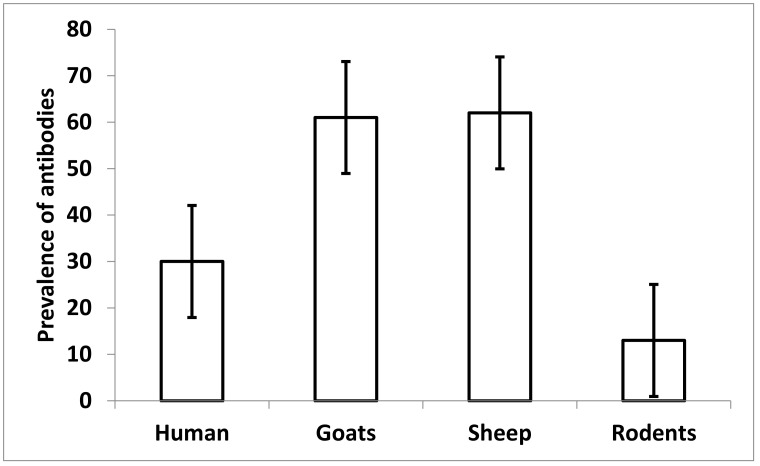
Proportion of leptospiral antibodies in humans, rodents and domestic animals (goats and sheep) in the semi-arid area in central Tanzania.

Results show that goats and sheep had the highest proportion of seropositivity for leptospiral antibodies, followed by humans and rodents (Figure 2). Goats and sheep are kept within a fenced homestead, which increases human–animal interaction and potential transmission of this zoonotic disease in this semi-arid area of central Tanzania.

**Figure 3:**
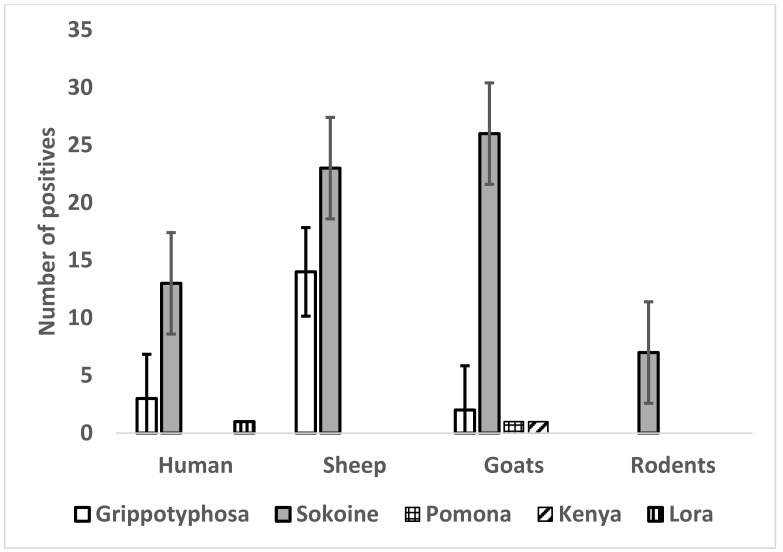
Occurrence of antibodies against *Leptospira* serovars in different hosts.

Antibodies against *Leptospira* serovar Grippotyphosa were detected in humans, sheep and goats, whereas serovar Sokoine was detected across species, including humans, sheep, goats and rodents (Figure 3). Serovar Pomona and Kenya were detected in goats only, and serovar Lora was detected in one human sample (Table 2). Slight cross-reaction was observed in a few samples that reacted to both serovar Grippotyphosa and serovar Sokoine in humans, sheep and goats.

### 2.4. Prevalence of Leptospira Seropositivity in Semi-Arid Setting versus Tropical Areas

The prevalence of *Leptospira* seropositivity in humans in the semi-arid region involving patients sampled in hospitals was higher (30%) than that reported in tropical regions of Tanzania (10.75%) [8,14]. This difference was statistically significant (χ^2^ = 14.636, df = 1, *p* = 0.0001). *Leptospira kirschneri* serovar Sokoine, which was the most prevalent in the two settings, was used to compare this prevalence. *Leptospira* seropositivity prevalence in goats and sheep from semi-arid areas (62%) was statistically significantly higher (χ^2^ = 11.577, df = 1, *p* = 0.0007) compared to 38% prevalence found in goats and sheep from Morogoro region [8] with bimodal rains. The difference in leptospiral antibody prevalence in rodents from the semi-arid area (13%) and previously reported in tropical Morogoro (17%) [8] was not statistically significant (χ^2^ = 0.361, df = 1, *p* = 0.5482). 

The prevalence of leptospiral antibodies in goats and sheep in the semi-arid setting (61–62%) was 1.6 times higher, nearly double, than the prevalence of leptospiral antibodies reported in goats in tropical areas (38%) (Table 3). Overall, human and animal leptospirosis burden in the semi-arid setting was higher than in the tropical settings. In the semi-arid setting, the prevalence of leptospiral antibodies in humans was 30% versus 10.75% in tropical areas; in goats and sheep it was 61 and 62%, respectively, versus 38% in tropical settings (Table 3).

Comparison of *Leptospira* seropositivity prevalence in the semi-arid setting and rainy/tropical regions of Tanzania, especially Morogoro region, which receives adequate bimodal rains per year, shows significant difference. Human and domestic animal *Leptospira* seropositivity prevalence was higher in semi-arid settings than in humid-rainy regions (*p* = 0.0001). Comparing the seropositivity of two key *Leptospira* serovars that are most prevalent in humans, goats, sheep and rodents in semi-arid areas, namely *Leptospira* serovar Sokoine (Table 3), followed by serovar Grippotyphosa, showed lower seropositivity in similar host species in the tropical region of Tanzania. In a previous study [7], the seropositivity of serovar Grippotyphosa in tropical settings in humans was as low as 0.5% versus 6% in this study, and 14% in sheep versus 25% found in this study. 

## 3. Discussion

Human and animal *Leptospira* seropositivity prevalence is higher in semi-arid areas than in rainy tropical regions reported previously in Tanzania [8]. High prevalence of leptospiral antibodies in human, goats and sheep in semi-arid settings indicates high transmission rate in the semi-arid areas, likely associated with frequent contacts of humans and animals at water sources that are scarcer in this semi-arid area [15]. The semi-arid area of Bahi central Tanzania lies in a depression of the great East Rift Valley where large numbers of livestock share the same source of water with humans undertaking various activities in the valley, including rice farming and grazing livestock [13,15]. Humans may be infected with spirochetes shed by infected animals as well as through contacts with animals kept in peridomestic pens. Leptospirosis has been reported in goats and sheep with higher prevalence in settings where goats and sheep are kept together [16]. *Leptospira interrogans* serovar Icterohaemorrhagiae has been reported in goats and sheep in Brazil, with potential transmission to humans [16]. 

In this study, *L. kirschneri* serovar Sokoine of the serogroup Icterohaemorrhagiae was the most prevalent among the five serovars tested. Serovar Sokoine was first isolated from cattle in Morogoro, eastern Tanzania [9] and has been most frequently reported in various animal species, including rodents [17], goats, sheep, dogs, cats, bats, freshwater fish, pigs and humans [8,10,11]. The high prevalence of *Leptospira* seropositivity in humans, goats and sheep observed in semi-arid setting in central Tanzania suggest potential high transmission rate from livestock to humans, especially involving *L. kirschneri* serovar Sokoine and serovar Grippotyphosa. *Leptospira* serovar Grippotyphosa has also been isolated from cattle in Morogoro, eastern Tanzania [8]. This serovar has been previously reported in Tanzania with low prevalence in humans and diverse animal host species, including goats, sheep, pigs, rodents, cats and dogs [8]. In this study, the prevalence of *L*. serovar Gripotyphosa in humans and livestock animals was also higher than those reported for this serovar in humid tropical Tanzania, suggesting a high transmission rate in semi-arid areas. 

The observed lower titer of seropositive animals suggested prolonged exposure of humans and livestock to *Leptospira* pathogens, where it is expected that animals which have been exposed to these pathogens for long period of time, such as a year, should demonstrate lower antibody levels than those with active infection indicated by higher antibody levels against the new infections. Further studies are needed to determine diversity and distribution of *Leptospira* serovars in different regions for successful management of this highly neglected zoonotic disease. It is noted that some of the *Leptospira* serovars commonly found in tropical Morogoro region were not detected in the semi-arid region. For example, antibodies against *L. borgepetersenii* serogroup Ballum serovar Kenya that is widespread in humans, bats, freshwater fish and domestic animals in eastern Tanzania [8,10,11] was not detected in this semi-arid area.

This study suggests potential role of livestock as maintenance hosts for leptospiras and a possible source of human leptospirosis transmission [10,11]. Large populations of cattle, sheep and goats are brought to the swampy areas for grazing and drinking water. Infected animals sheds leptospiras into the water sources and swamps, which promotes wide transmission to humans and other animals that share the water resource. Vaccination of livestock animals against leptospirosis using local strains-based vaccines can significantly reduce the transmission of leptospirosis to livestock animals and ultimately to humans, especially livestock keepers in these areas, where human–animal interaction around water sources is high. In this study, higher prevalence of antibodies against *Leptospira* serovar Grippotyphosa was found to prevail in humans, goats and rodents. *Leptospira* serovar Sokoine was dominant in these hosts and rodents, suggesting potential sharing of a transmission source. The fact that goats and sheep are kept within human settlements points to a potential source of leptospirosis transmission to humans.

Seropositivity in rodents was slightly lower (15.5%) in the semi-arid setting than the previously reported prevalence of 17% in bimodal rainy regions [8]. The majority of rodents captured in this study were *Mastomys* spp., which are the most abundant rodent species in this region, but they carried a relatively small proportion of *Leptospira* seropositivity likely due to their ecology and habitats, which differ from that of indicator rodent species, namely the African giant pouched rats (*Cricetomys* spp.) and insectivores shrews (*Crocidura* spp.), which are reported to carry more burden of leptospiras, associated with their ecology [8]. However, comparing this finding with another study in the tropical Morogoro town involving a large sample set of 500 rodents with leptospiral antibody prevalence of 5% [14] showed significant difference in prevalence between the semi-arid and tropical areas (15.5% versus 5%). This difference is probably influenced by small sample size collected in the unique Bahi depression which is likely to confine rodents to the valley and hence increase circulation of leptospiral transmission within the population, yielding prevalence that is higher than that of a larger sample from wider geographic areas of tropical Morogoro [14]. On the other hand, the present findings, although derived from a relatively small sample strategically collected from the Bahi depression, including rice fields, may represent the actual rodent situation in the study area, such that increasing the sample size may still yield the same results, since the few captured rodents and shrews represented a wider geographic coverage within the Bahi rift valley or depression. This is further supported by a previous report of *Leptospira* seropositivity prevalence of 17% from a study involving 90 rodents [14]. 

The population of rodents in the study area was low, as shown by few captures despite of large number of traps and traps nights spent during the study period. The low proportions of seropositive rodents among the few captured animals suggest that they probably were a migrant population after flooding that likely killed the native or resident rodents. This assumption may be supported by the uncommonly large number of embryos in gravid females captured in the rice fields in this valley. The high number of embryos is probably an instinctive mechanism to repopulate the valley after the floods. The low trapping success was, therefore, a limitation of this study. A subsequent study some months later would have achieved a higher trapping success, based on the high number of embryos demonstrated in the gravid females. A larger sample size would give more insight and understanding of the prevalence of leptospirosis in the rodents to compare previous findings from a study with a larger sample size of rodents [14]. Seasonal sampling is therefore recommended to understand the carrier role of rodents with respect to leptospirosis. 

## 4. Materials and Methods

This cross-sectional study involved sampling humans and animals in a semi-arid setting of Tanzania.

### 4.1. Study Site

The study was conducted in Bahi District, central Tanzania (S 05°58.9′ E 035°18.473′ with elevation of 826 m above sea level). Bahi is a semi-arid area with a short annual rain season (December to March). Sampling was carried out after the rainy season from end of March to April 2016. Rodent trapping was conducted in rice fields along the river flowing into Lake Sulunga or Bahi swamp at Nagulo Bahi village (S 06°00.961′ E 035°14.390′ with elevation of 820 m above sea level) and in houses bordering the rice fields at Bahi Sokoni village. 

Bahi district has semi-arid type of climate, experiencing one rainfall in a season, which is mostly erratic. The drainage is characterized by seasonal rivers and swamps/wetlands with few permanent rivers. Seasonal and permanent water resources in Bahi district are the major source of water for domestic use, animals and irrigation [15]. 

Water and pasture for livestock is scarce in Bahi district, especially in the dry season, which increases interaction with humans at available water sources. Animals sharing water with humans at the scarcer water sources [15] can contaminate the water with their urines and feces which enhances transmission of water borne infections to humans. In the dry season, livestock keepers walk up to 7 km daily for grazing their animals, for example from Nagulo Bahi village to Lake Sulunga or Bahi plains, also known as Bahi swamps. 

### 4.2. Socioeconomic Status, Risky Occupation and Diseases in the Study Area

Nearly 80 percent of the study area economy is from farming, mostly by smallholder farmers growing maize, sorghum, bulrush millet, groundnuts, sunflower, paddy, sweet potatoes and cassava. Livestock keeping is also a major economic activity, whereby 28% of the population keep cattle, goats (37%), sheep (19%), chickens (78%) and donkey (<10%) [15], according to the Dodoma animal census of 2008. The major 10 diseases reported in the study area include malaria, acute respiratory infections (ARI), diarrhea, helminthiasis, ear infections, eye infection, pneumonia, skin infection, urinary tract infection and noninfection [15]. Malaria is the leading disease followed by ARI [15]. Like in many countries across Africa, leptospirosis disease is not diagnosed in Tanzania and is easily misdiagnosed to malaria and other diseases with similar symptoms and signs. In northern Tanzania, leptospirosis is reported in 20 percent of patients with nonmalarial fevers [6].

### 4.3. Human Sampling

Human *Leptospira* seropositivity was surveyed in collaboration with health facilities in the study area. Blood samples were obtained from patients visiting the health facilities for various diagnostic tests, including malaria and typhoid, after consenting. Demographic information, including gender and age of participants, was recorded. 

### 4.4. Inclusion and Exclusion Criteria for Human Subjects

The study included adult human participants residing within the study locality with fevers and seeking laboratory diagnosis. Individuals under 18 years old and those who did not agree to consent were excluded from the study. No individual interviews were conducted with participants to ascertain whether they had history of signs related to leptospirosis because they are related to commonly known diseases such as malaria.

### 4.5. Rodents and Other Small Mammals Trapping

Sampling of rodents and shrews (insectivores) was done on rice fields and along the river and wetland areas using Sherman live rodent traps (H.B. Sherman, Tallahassee, FL, USA). The traps were set inside the rice fields and along rice field ridges that have natural vegetation cover suitable for rodent refuge and which become safe elevated habitats for the rodents during flash flooding. 

The traps were baited with peanut butter mixed with maize flour. Dried Lake Victoria sardine fish (*Rastrineobola argentea*) were also used to attract insectivores (shrews). Traps were set for a minimum of three days (syn. trap nights) per site before shifting to another location. A total of 200 traps were set in the area at five meters apart. Traps were checked each morning to collect captured animals. The trapped animals were humanely anaesthetized with diethyl ether-soaked cotton wool before being processed. External morphological measurement including body weight, length of tail, head and body and the ear were recorded for primary identification and estimation of age of the animals. Blood was collected from the supraorbital vein or by heart puncture and was left to settle/clot and separate the serum at room temperature. Serum samples were stored at −20 °C until used in serological microscopic agglutination test (MAT) [18,19] for *Leptospira* antibodies. 

### 4.6. Sheep and Goats Sampling

Villagers were informed about the study, and 6 major livestock keepers who reported to graze their animals from the village to the nearby and only water source, Lake Bahi, agreed to have their goats and sheep sampled, and rodents trapped around their homestead. The homesteads of the livestock keepers who consented and were selected for this study were distributed across the village and hence were representative of the other livestock keepers not included in the study. The 2006 district livestock census reported a total of 620 goats and 151 sheep at Nagulo Bahi village [15] where sampling was conducted for this study. A minimum of 7 goats and sheep were sampled from each of the consenting participants to obtain a minimum of 42 goats and sheep for this study, which is a 7 and 20 percent representation of the animal’s population, respectively. Sampling was done in the morning hours before animals were taken for grazing and in the grazing areas. Blood was aseptically collected from the jugular vein into a plain vacutainer tube. Blood was allowed to clot, and serum was separated for serological detection of *Leptospira* antibodies. 

### 4.7. Microscopic Agglutination Test (MAT)

Antibodies against *Leptospira* were determined by MAT as previously described [18,19]. *Leptospira* serovars used as antigens were selected from recently proposed candidate antigens for MAT in humans and animals [8]. These included *Leptospira kirschneri* serogroup Grippotyphosa serovar Grippotyphosa, *L. kirschneri* serogroup Icterohaemorrhagiae serovar Sokoine, *L. interrogans* serogroup Australis serovar Lora, *L. borgpetersenii* serogroup Ballum serovar Kenya and *L. interrogans* serogroup Pomona serovar Pomona. Cultures were prepared in Ellinghausen McCullough medium, modified by Johnson and Harris (EMJH), containing 5-fluorouracil as selective inhibitor. Well-grown cultures with density of approximately 300 × 10^8^ leptospires/mL, obtained after 5 days of incubation at 30 °C were used as live antigen for the MAT. Serum samples were initially serially diluted in phosphate buffered saline (pH 7) starting with 1:10, 1:20, 1:40 and 1:80 that was mixed with 50 μL of live *Leptospira* antigen that doubled the serial dilution to 1:20, 1:40, 1:80 and 1:160 recommended for initial screening. Considering that the study area is a potential endemic area, all positive samples that reacted with any of the test antigens at ≥1:20 were further titrated to a final dilution of 1:20,480 to determine the end titer or the antibody level, which informed whether the infection was recent or passive. This was especially important, since the human subjects were individuals visiting hospitals with suspected illness requiring blood test, while animals were asymptomatic. The MAT readings of the titration test were used in subsequent analysis.

### 4.8. Data Analysis

The *Leptospira* seropositivity prevalence in humans and animals from the study area was compared with data from humid tropical (non-semi-arid) areas, obtained in previous studies, and the differences in *Leptospira* seropositivity prevalence between the humans and animals from the two settings were assessed by Pearson χ^2^. Statistical analyses were conducted using MedCalc [20,21]. Findings were considered statistically significant at *p* ≤ 0.05. 

### 4.9. Ethical Consideration

The study on humans was approved by the Medical Research Coordinating Committee of the National Institute for Medical Research and granted ethical clearance certificate No. NIMR/HQ/R.8a/vol.IX/2453. 

Permission was also sought from local authorities in the study area. Adult human participants visiting hospitals in the study areas were informed about the study, and those who volunteered gave informed oral consent allowing anonymous screening for leptospirosis in addition to primary blood tests for which they were recruited. Since leptospirosis is not known and not routinely diagnosed and is considered among causes of nonmalaria fevers, the ethical committee approved use of blood samples from anonymous participants, especially with nonmalaria fevers. Hence, written consent was not available for retrospective anonymous participants whose samples were available for this study. The Ethical Review Board of Sokoine University of Agriculture approved the use of oral consent documented by the health facility as “consented orally.” The institutional review board also approved the use of animals in this study, whereas the permit for capture and use of wild rodents was granted by the Tanzania Commission for Science and Technology (COSTECH), permit no. 2013-260-NA-2014-110. Animals were handled in compliance with the “Animal Research: Reporting In Vivo Experiments” (ARRIVE guidelines) [22].

## 5. Conclusions

In conclusion, this study showed high *Leptospira* seropositivity prevalence suggest that leptospirosis could be a major public health and animal health problem that needs appropriate attention even in semi-arid areas of Tanzania. Awareness of leptospirosis disease among medical communities and general public is much needed. Awareness should include enhancing teaching leptospirosis in medical schools and clinical colleges through supporting availability of *Leptospira* teaching materials and darkfield microscopes for examination of leptospires. A semi-arid setting that characteristically has increased humans and animals’ interactions at water sources have high risk of leptospirosis transmission therefore control intervention such as vaccination of animals with local strain-based vaccines should be considered to these settings. This can be achieved by further researching on the locally circulating *Leptospira* strains to determine whether they are covered in vaccines available elsewhere. Rapid leptospirosis diagnostic test kits, although they are not very reliable, are needed for rapid testing of the disease in humans, especially when other causes of fevers are ruled out [23]. Real-time PCR (RT-PCR) should be considered for quick and reliable detection of leptospiral DNA in patients with fevers [24,25]. Although such molecular tests may not precisely inform about identity of the local infecting *Leptospira* serovar, the information needed for pinpointing sources of infection, but the ability to produce rapid findings with these methods enables timely management of leptospirosis in patients than the gold standard MAT that takes longer time. None of the participating facility that provided blood samples had diagnostic kits for leptospirosis screening, although reports show that about 20% of patients with nonmalarial fevers [6] and risk population groups of sugarcane plantation workers and fishing communities in northern Tanzania have leptospirosis [26]. Isolation and identification of infecting serovars should be emphasized along serodiagnosis to better understand the prevalent serovars for appropriate control strategies. Similar studies are needed in other semi-arid settings to obtain insights on the burden of this disease which is highly neglected in Africa and elsewhere and can be a major cause of fevers of unknown origins.

## Figures and Tables

**Table 1:** Composition and percentage of leptospiral antibody titer in different host species.

Host	1:20	1:40	1:80	Total Positive
Human	13 (86%)	2 (13%)	0	15
Sheep	20 (58.8%)	14 (41.2%)	0	34
Goats	15 (53.5%)	11 (39.3%)	2 (7.1%)	28
Rodents and shrews	6 (85.7%)	1 (14.3%)	0	7

**Table 2:** *Leptospira* serovar antibodies detected in different hosts included in microscopic agglutination test (MAT).

Host	Leptospira Serovars				
	Grippotyphosa	Sokoine	Pomona	Kenya	Lora
Human	3 (1) *	13 (1) **	0	0	1
Sheep	14 (3) *	23 (3) **	0	0	0
Goats	2 (2) *	26	1	1	0
Rodents	0	7	0	0	0

* cross-reaction with serovar Sokoine. ** cross-reaction with serovar Grippotyphosa.

**Table 3:** Comparison between *Leptospira* seropositivity prevalence in humans and animals from semi-arid versus tropical areas.

	Number of Participants		Seropositivity (%)		Seropositivity between Localities (*p* ≤ 0.05)
Species	Semi-Arid	Tropical	Semi-Arid	Tropical	
Humans	50	400	30	10.75*	*p* = 0.0001
Goats and sheep	102	100	62	38*	*p* = 0.0007
Rodents	45	90	15.5	17*	*p* = 0.5482

* Source [8].

## Data Availability

Data supporting reported results can be available from SPMC source on request.
